# Metabolic alterations in acute myocardial ischemia-reperfusion injury and necrosis using *in vivo* hyperpolarized [1-^13^C] pyruvate MR spectroscopy

**DOI:** 10.1038/s41598-019-54965-7

**Published:** 2019-12-05

**Authors:** Chung-Man Moon, Yun-Hyeon Kim, Young-Keun Ahn, Myung-Ho Jeong, Gwang-Woo Jeong

**Affiliations:** 1 Quantitative Medical Imaging Section, National Institute of Biomedical Imaging and Bioengineering, National Institutes of Health, Bethesda, MD USA; 2 Research Institute of Medical Sciences, Chonnam National University, Gwangju, Republic of Korea; 3 Department of Radiology, Chonnam National University Medical School, Gwangju, Republic of Korea; 4 Department of Cardiology, Chonnam National University Medical School, Gwangju, Republic of Korea

**Keywords:** Diagnostic markers, Biophysical chemistry

## Abstract

This study aimed to investigate real-time early detection of metabolic alteration in a rat model with acute myocardial ischemia-reperfusion (AMI/R) injury and myocardial necrosis, as well as its correlation with intracellular pH level using *in vivo* hyperpolarized [1-^13^C] pyruvate magnetic resonance spectroscopy (MRS). Hyperpolarized ^13^C MRS was performed on the myocardium of 8 sham-operated control rats and 8 rats with AMI/R injury, and 8 sham-operated control rats and 8 rats with AMI-induced necrosis. Also, the correlations of levels of [1-^13^C] metabolites with pH were analyzed by Spearman’s correlation test. The AMI/R and necrosis groups showed significantly higher ratios of [1-^13^C] lactate (Lac)/bicarbonate (Bicar) and [1-^13^C] Lac/total carbon (tC), and lower ratios of ^13^C Bicar/Lac + alanine (Ala), and ^13^C Bicar/tC than those of the sham-operated control group. Moreover, the necrosis group showed significantly higher ratios of [1-^13^C] Lac/Bicar and [1-^13^C] Lac/tC, and lower ratios of ^13^C Bicar/Lac + Ala and ^13^C Bicar/tC than those of the AMI/R group. These results were consistent with the pattern for *in vivo* the area under the curve (AUC) ratios. In addition, levels of [1-^13^C] Lac/Bicar and [1-^13^C] Lac/tC were negatively correlated with pH levels, whereas ^13^C Bicar/Lac + Ala and ^13^C Bicar/tC levels were positively correlated with pH levels. The levels of [1-^13^C] Lac and ^13^C Bicar will be helpful for non-invasively evaluating the early stage of AMI/R and necrosis in conjunction with reperfusion injury of the heart. These findings have potential application to real-time evaluation of cardiac malfunction accompanied by changes in intracellular pH level and enzymatic activity.

## Introduction

Cardiovascular diseases remain the leading cause of mortality and morbidity worldwide^1^. Of these, myocardial ischemia occurs when the oxygen supply to the myocardium is insufficient^2^. Increasing severity of ischemia leads to progressive changes, including diastolic heart failure, left ventricular systolic dysfunction, and electrographic disturbance^3^. More severely, ischemia of prolonged duration induces myocardial infarction (MI) which causes irreversible myocardial injury resulting in necrosis of a significant portion of myocardium^4^. Moreover, myocardial reperfusion injury adds tissue damage to the infarct region. Early reperfusion is the goal of therapy in patients with ST-elevation myocardial infarction because the reopening of an acutely occluded coronary artery reduces the size of the myocardial infarction. But this approach is associated with transient contractile dysfunction, arrhythmias, metabolic acidosis, and increased release of myocardial enzymes, which paradoxically can compound the cellular metabolic changes that occur in ischemia^4^. Therefore, early detection of the acute metabolic alterations that occur during ischemia-reperfusion injury and necrosis may further our understanding of the underlying mechanisms of cardiac disease and would aid in the development of new clinical diagnostic strategies in patients with acute myocardial infarction undergoing acute revascularizations.

With the help of electrocardiography (ECG)^5^ and magnetic resonance imaging (MRI)^6^, ischemia can be detected indirectly by electrical changes, relative changes in perfusion, or abnormal myocardial function in patients with coronary artery disease. Positron emission tomography (PET) has also been used to measure perfusion and accumulation of a tracer. However, it is unable to quantify the downstream metabolites derived from the tracer^7^.

In the past decade, solution-state hyperpolarized ^13^C magnetic resonance spectroscopy (MRS) and imaging have offered the possibility to noninvasively and very rapidly assess cellular metabolism *in vivo*^8^. To overcome the low signal-to-noise ratio (SNR), ^13^C-labeled substrates that have undergone dynamic nuclear polarization (DNP) produce ^13^C signals with 10,000-fold enhancement compared to thermal polarization levels. A major advantage of ^13^C labeling is the extended range of the chemical shift range of 250 ppm compared with the ^1^H spectrum (15 ppm), which yields a much higher spectral resolution^9^.

Hyperpolarized ^13^C-metabolic MRS provides the possibility to study rapid changes in metabolism during myocardial reperfusion, enabling *in vivo* analysis of myocardial injury^7,10,11^. In hyperpolarized MR studies of the heart, pyruvate (Pyr) is important given its central role at the crossroad between glycolysis and oxidative phosphorylation, in which [1-^13^C] Pyr is converted via enzyme-mediated reactions to [1-^13^C] lactate (Lac), [1-^13^C] alanine (Ala), and ^13^C bicarbonate (Bicar)^7^. Hyperpolarized MR imaging can be used to investigate substrate metabolism in both the isolated perfused rat heart^11^ and the *in vivo* heart^12^ after myocardial ischemia.

In many biochemical processes, detecting pH *in vivo* and noninvasively is important because changes of pH are associated with pathologies in biological systems, such as cancer, inflammation, infection, and ischemia^13^. Hyperpolarized ^13^C-metabolic MRS can be used to directly measure flux through pyruvate dehydrogenase (PDH) with the formation of CO_2_ and HCO_3_^−^ (Bicar), showing the corresponding pH information^14,15^. Hyperpolarized ^13^C-labelled Bicar is one of the methods for pH measurement by determination of the CO_2_/HCO_3_^−^ ratio^16^. Although a few hyperpolarized ^13^C MRS studies^7,17^ have reported changes in metabolites in an animal model of myocardial ischemia-reperfusion, to the best of our knowledge, an *in vivo* MRS study has not yet been done of hyperpolarized [1-^13^C] Pyr focusing on differential metabolic changes in both acute myocardial ischemia-reperfusion (AMI/R) injury and necrosis with different pH values for further clinical translation.

The purpose of this study was therefore to investigate metabolic alterations associated with AMI/R injury and necrosis and to assess their correlation with intracellular pH levels in the myocardium through the use of *in vivo* hyperpolarized ^13^C MRS.

## Methods

### Ethics

This study was approved by the Institutional Animal Care and Use Committee of Chonnam National University. All experiments were performed in accordance with the relevant guidelines and regulations.

### Animal model

Eighteen pathogen-free, 7-week-old, male Sprague-Dawley rats were received from Orient Co. (Iksan, Korea). The rats were housed in an automatic temperature controlled environment with a 12-h light-dark cycle. During surgery, rats were anaesthetized with 2.5–3.0% isoflurane, intubated, and mechanically ventilated at a rate of 60 breaths per minute with a small-animal ventilator (Harvard Rodent Ventilator; Harvard Apparatus, South Natick, MA, USA)^18,19^. Eighteen rats that underwent left thoracotomy without left anterior descending (LAD) coronary artery occlusion were used as sham-operated controls. Then, these rats underwent the same protocol but with LAD coronary artery occlusion in the myocardium using a curved needle and 5-0 silk, according to guidelines for experimental models of myocardial ischemia and infarction^20,21^. In order to facilitate suture removal, a small silicon ring was inserted in the silk thread below the knot. Then, the chest was closed. After 20 min for 8 rats and 90 min for 8 rats, reperfusion was initiated by reopening the chest and cutting the ligature around the LAD coronary artery. Two sham-operated rats were used in histopathologic analysis. At the end of the experiments, all animals were euthanized according to the guidelines of the Animal Veterinary Medical Association.

### Animal preparation

As described previously^18,19^, the rats were anesthetized with 2.5–3% isoflurane mixed with oxygen (1 L/min). A 24-gauge catheter was inserted into the tail vein of each rat for intravenous (IV) administration of the hyperpolarized [1-^13^C] Pyr solution. During MRI and ^13^C MRS measurements, the breathing rate of the rat was monitored with a model 1025 pressure transducer (SA Instruments Inc., Stony Brook, NY, USA) and was maintained between 60 and 70 breaths/min. Body temperature was also monitored with a non-magnetic rectal temperature probe (FISO Technologies, Quebec City, Quebec, Canada) and was kept at approximately 36 °C.

### Polarization and hyperpolarized ^13^C MR spectroscopy

The protocol was decribed previously in our published papers^18,19^. [1-^13^C] pyruvic acid (23–24 mg; Isotec, Miamisburg, OH) containing 15 mM of trityl radical (OX063; Oxford Instruments, Abingdon, UK) was transferred to a polytetrafluoroethylene sample cup and frozen using liquid helium^18,19^. The pyruvic acid sample was hyperpolarized using the Hypersense DNP polarizer (Oxford Instruments) at 3.35 T and 1.4 K with 94.1 GHz microwave irradiation for 1–1.5 h, then dissolved in 4.2–4.3 mL of superheated alkaline buffer containing 40 mM Trizma Pre-Set Crystals pH 7.6, 80 mM NaOH, 50 mM NaCl, and 2.69 mM disodium EDTA^18,19^. The hyperpolarized [1-^13^C] Pyr solution (80 mM) was intravenously injected through a catheter placed in the tail vein of the rat^18,19^. An aliquot of the solution was used to measure the percent polarization (17–20%) using a MQC spectrometer (Oxford Instruments) and the pH (7.0–7.5) was measured using an AUW-220D electronic balance (Shimadzu, Tokyo, Japan)^18,19^.

Hyperpolarized ^13^C MRS was performed on a 3 T GE MR750 scanner (GE Healthcare, Milwaukee, WI, USA) using a custom-built surface coil with an inner diameter of 37 mm^18,19^. For the dynamic study, ^13^C spectra were acquired every 2 s for 120 s just after injection of Pyr from a 10-mm slice with a 70 × 70-mm field of view (FOV) on the heart at 15 min of reperfusion following 20 and 90 min of coronary artery occlusion. Prior to hyperpolarized ^13^C study, the transmit gain was calibrated using a thermally polarized ^13^C‐enriched urea phantom (8 M, doped with Magnevist 1% by volume) placed near center of the coil. Dynamic MRS data were acquired using a free induction delay chemical shift imaging (FIDCSI) pulse sequence (GE Healthcare) with a 5000 Hz acquisition bandwidth, 4096 points, and a slice-selective radiofrequency pulse with 10° flip angle^18,19^. During the pre-scan period prior to acquiring ^13^C spectra, linear shimming was performed over the cardiac tissue. The rats underwent MRI scan with a length of approximately 10 min, followed by 2 min of hyperpolarized ^13^C MRS with [1-^13^C] Pyr. ^13^C dynamic spectra were acquired from 8 sham-operated control rats and 8 rats with AMI/R injury, and 8 sham-operated control rats and 8 rats with AMI-induced necrosis.

### Data processing and statistical analysis

MR spectra were post-processed and analyzed using SAGE software (GE Healthcare), as described previously by Moon *et al*.^18,19,22^. Dynamic MRS data were apodized with a 9-Hz Gaussian filter in the time domain, and zero-filled to 16,384 data points followed by Fourier transformation. The dynamic spectra in absorption mode were baseline corrected followed by phase correction at zero and first orders^18,19,22^. The peak heights of metabolite signals were used for the quantification of [1-^13^C] Lac (185 ppm), [1-^13^C] Pyr-hydrate (181 ppm), [1-^13^C] Ala (177 ppm), [1-^13^C] Pyr (172 ppm), ^13^C Bicar (162 ppm), and ^13^CO_2_ (126 ppm)^14,18,19,22^. The individual metabolites were normalized to total carbon (tC) signals, which is the sum of the [1-^13^C] Lac, [1-^13^C] Pyr-hydrate, [1-^13^C] Ala, [1-^13^C] Pyr, ^13^C Bicar, and ^13^CO_2_ signals. In addition, the ^13^C Bicar metabolite was normalized to the [1-^13^C] Lac + Ala signals, and the [1-^13^C] Lac metabolite was normalized to the ^13^C Bicar signal^7,17^. For quantification of the exchange rates, the area under the curve (AUC) ratios of hyperpolarized [1-^13^C] Lac to [1-^13^C] Pyr (Lac/Pyr) and [1-^13^C] Bicar to [1-^13^C] Pyr (Bicar/Pyr) were calculated in three groups^23^. The differential metabolite ratios between the two groups were analyzed by Wilcoxon signed-ranks test and Mann-Whitney U-test at P < 0.05 (two-tailed test) using SPSS 19.0 (Chicago, IL, USA)^19^. Percent changes (%) in metabolite level by AMI/R injury and necrosis were calculated by the following equation^19^:

$${\rm{Metabolite}}\,{\rm{change}}\,({\rm{MC}})\,( \% )={({\rm{MC}}}_{{\rm{after}}{\rm{AMI}}/{\rm{R}}{\rm{or}}{\rm{necrosis}}}\mbox{--}{{\rm{MC}}}_{{\rm{sham}}})/{{\rm{MC}}}_{{\rm{sham}}}\times 100$$

*In vivo* intracellular pH value was regulated by a Bicar buffer system. The pH calculation with the Henderson-Hasselbalch equation is allowed by pH-dependent chemical equilibrium of ^13^C Bicar and ^13^CO_2_^16^. As a dissociation constant, pKa is assumed to be 6.17 *in vivo*^16^. Since the peak heights of maximal signal intensity of hyperpolarized ^13^C-labeled Bicar and formed CO_2_ are proportional to molecular concentrations, pH was calculated by the ratio of these signals. Also, Spearman’s correlation test was used to analyze the correlations of [1-^13^C] metabolite levels and pH values with a 95% confidence interval. In addition, a receiver operating characteristics (ROC) curve was analyzed to assess the diagnostic performance of hyperpolarized [1-^13^C] pyruvate MRS in the sham-operated control, AMI/R, and necrosis groups.

### Serum biochemical analysis

After hyperpolarized ^13^C MRS study, blood samples of 0.5–1 mL were collected from all rats. Serum samples were collected by centrifugation at 3,000 rpm for 15 min and stored at −80 °C until used for biochemical analyses, as described in previous studies^18,19^. Creatine kinase (CK), aspartate aminotransferase (AST), and lactate dehydrogenase (LDH) were measured using an autoanalyzer with commercially available test kit (VET TEST 8008; IDEXX Laboratories, Westbrook, ME, USA)^18,19^.

### Histopathological analysis and triphenyltetrazolium staining

Myocardial tissue fragments of approximately 1.4 × 1.2 × 0.3 cm^3^ were extracted from sham-operated control and AMI/R-induced rats and fixed in 10% formaldehyde. After 24 h fixation, the fragments were dehydrated, cleared, and embedded in paraffin^18,19^. Then, paraffin blocks were cut into 2 μm-thick sections and stained by hematoxylin and eosin (H&E) to assess cardiac damage^18,19^. A digital camera (FO124SC; FOculus, Finning, Germany) used to acquire the slice images for histopathology assessment.

Also, after hearts were cut into 2-mm transverse slices, tissue slices were incubated in 2% 2,3,5-triphenyltetrazolium chloride (TTC) for 10 min at 37 °C to allow the demarcation of the infarct region.

## Results

### Body weight

There were no significant differences in body weights (g) between sham-operated control rats and rats with AMI/R injury, and sham-operated control rats and rats with AMI-induced necrosis (319.5 ± 13.6 vs. 317.0 ± 11.7 and 318.1 ± 10.4 vs. 316.2 ± 9.8, respectively).

### Histologic and enzymatic changes after AMI/R injury and necrosis

**Figure 1:**
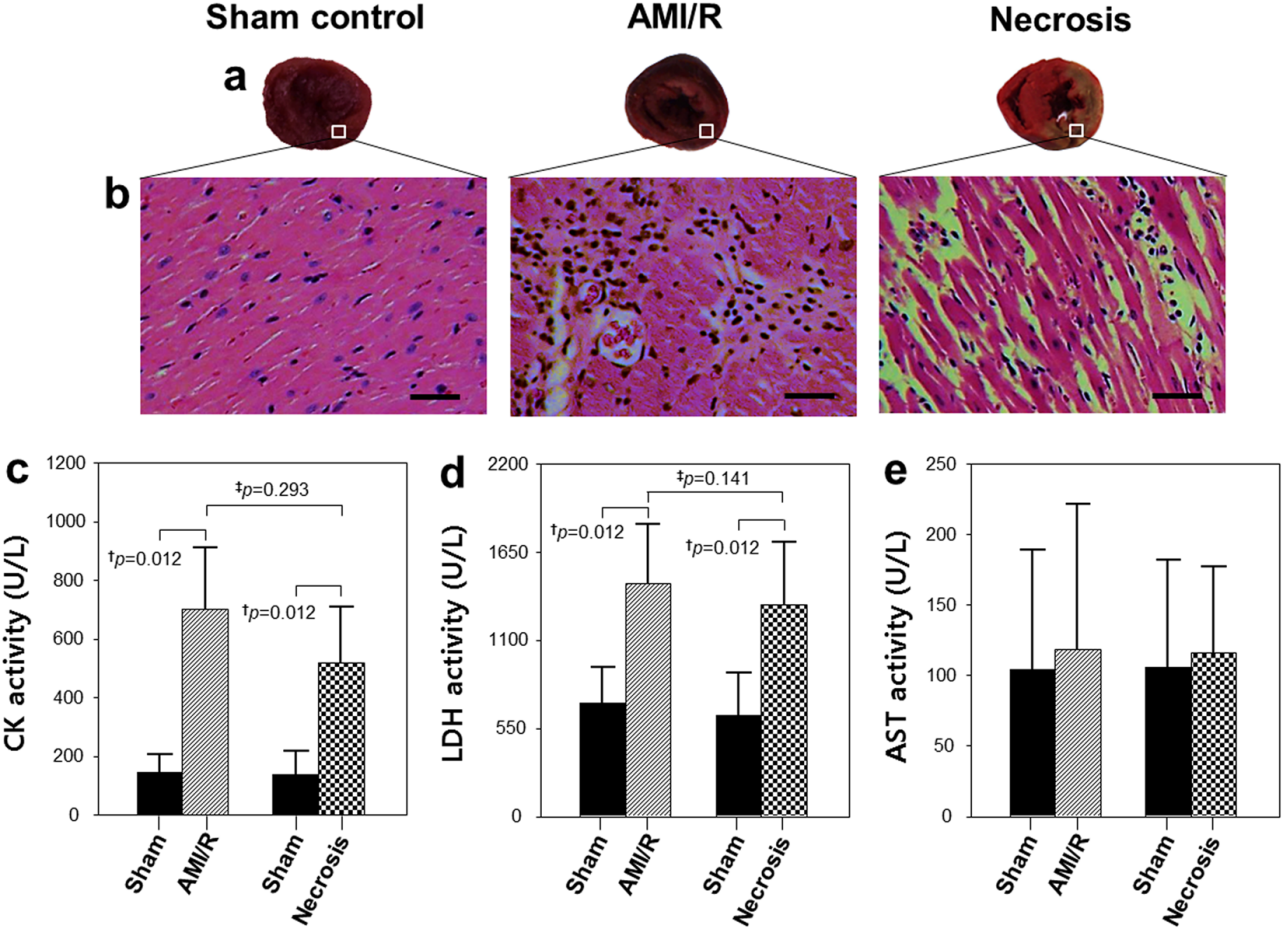
Myocardium stained with TTC. (**a**) Representative hematoxylin and eosin (H&E) staining results for myocardial sections (magnification, ×200). (**b**) Enzymatic activities of creatine kinase (CK) (**c**), lactate dehydrogenase (LDH) (**d**), and aspartate aminotransferase (AST). (**e**) ^†^Significant difference (Wilcoxon signed-rank test; P < 0.05) between rats before and after AMI/R, and between rats before and after necrosis; ^‡^significant difference (Mann-Whitney U test; P < 0.05) between AMI/R and necrosis groups. Bar = 50 μm.

TTC stains viable tissue a deep red color, whereas area of infarct due to necrosis remain unstained and thus appear white (Fig. 1a). Also, Fig. 1b shows typical H&E- stained myocardial tissues with normal architecture and pathologic changes. Compared with the sham-operated normal heart, a widened inter-fiber space was prominent after AMI/R, presumably because of the accumulation of interstitial fluid. Moreover, necrotic cardiac myocytes with loss of nuclei and less prominent striation were observed in infarcted myocardium, consistent with previous findings^24^. Consequently, the two model groups could be distinguished histologically: one with ischemic injuries similar to those of stunned or injured myocardium and the other with necrotic myocardium.

Levels of CK and LDH were significantly higher in the AMI/R and necrosis groups than in the sham-operated control group (Fig. 1c,d). However, there were no significantly different body weights and AST levels among the three groups (Fig. 1e).

### Quantification of metabolites in ^13^C dynamic MRS

**Figure 2:**
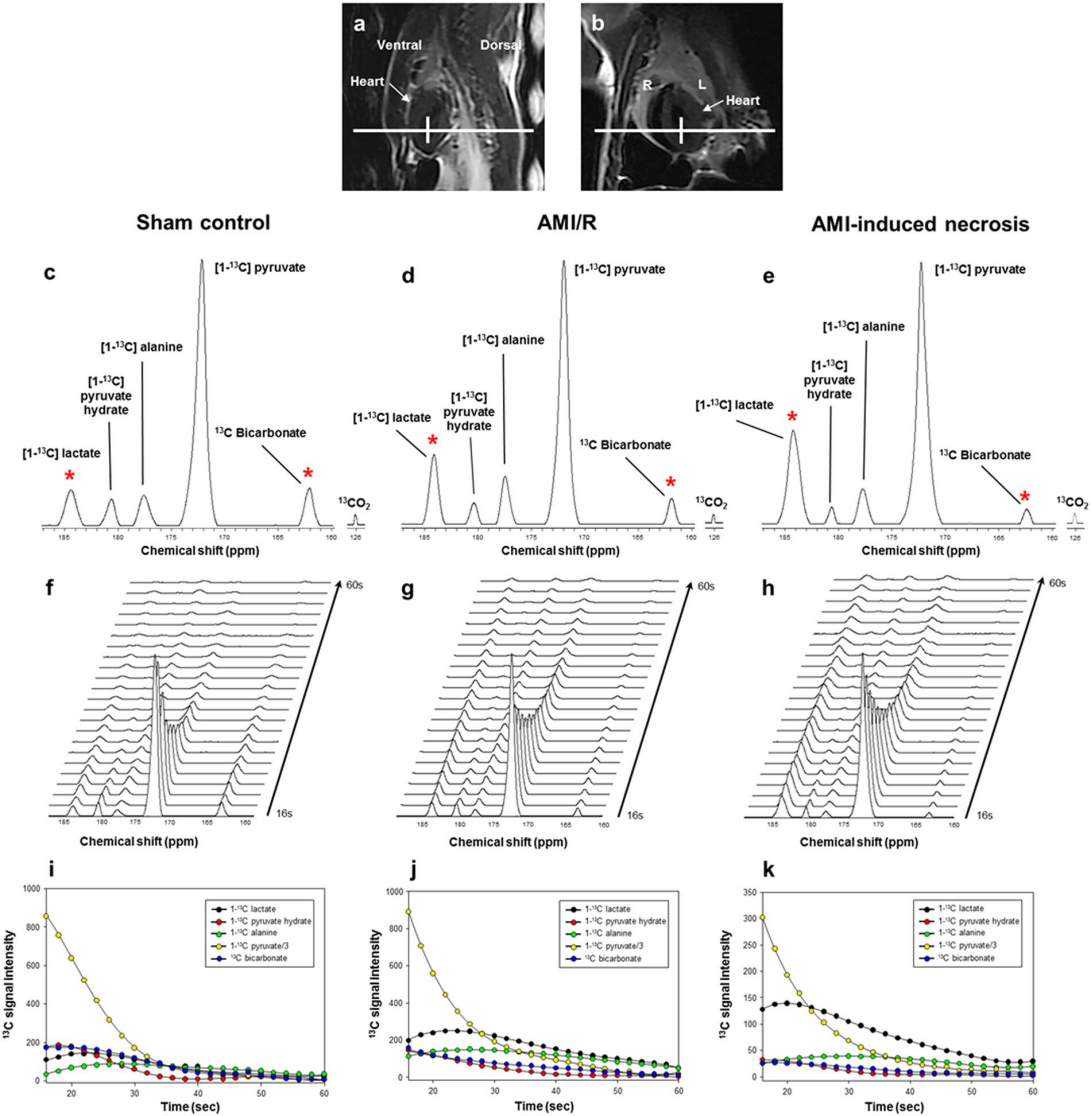
Dynamic ^13^C MR spectra of the heart (**a**: sagittal image; **b**: coronal image), which were acquired from sham-operated control rat (left), rat with AMI/R (middle), and rat with AMI-induced necrosis (right). Sum spectra (**c**–**e**) were reconstructed from the stack spectra (**f**–**h**) covering the 16- to 60-second time span. Signal intensity curves (**i**–**k**) demonstrate the time course of conversion of [1-^13^C] lactate, [1-^13^C] pyruvate-hydrate, [1-^13^C] alanine, [1-^13^C] pyruvate, and ^13^C bicarbonate. *Indicates the significantly different metabolite ratios between the groups (P < 0.05).

**Figure 3:**
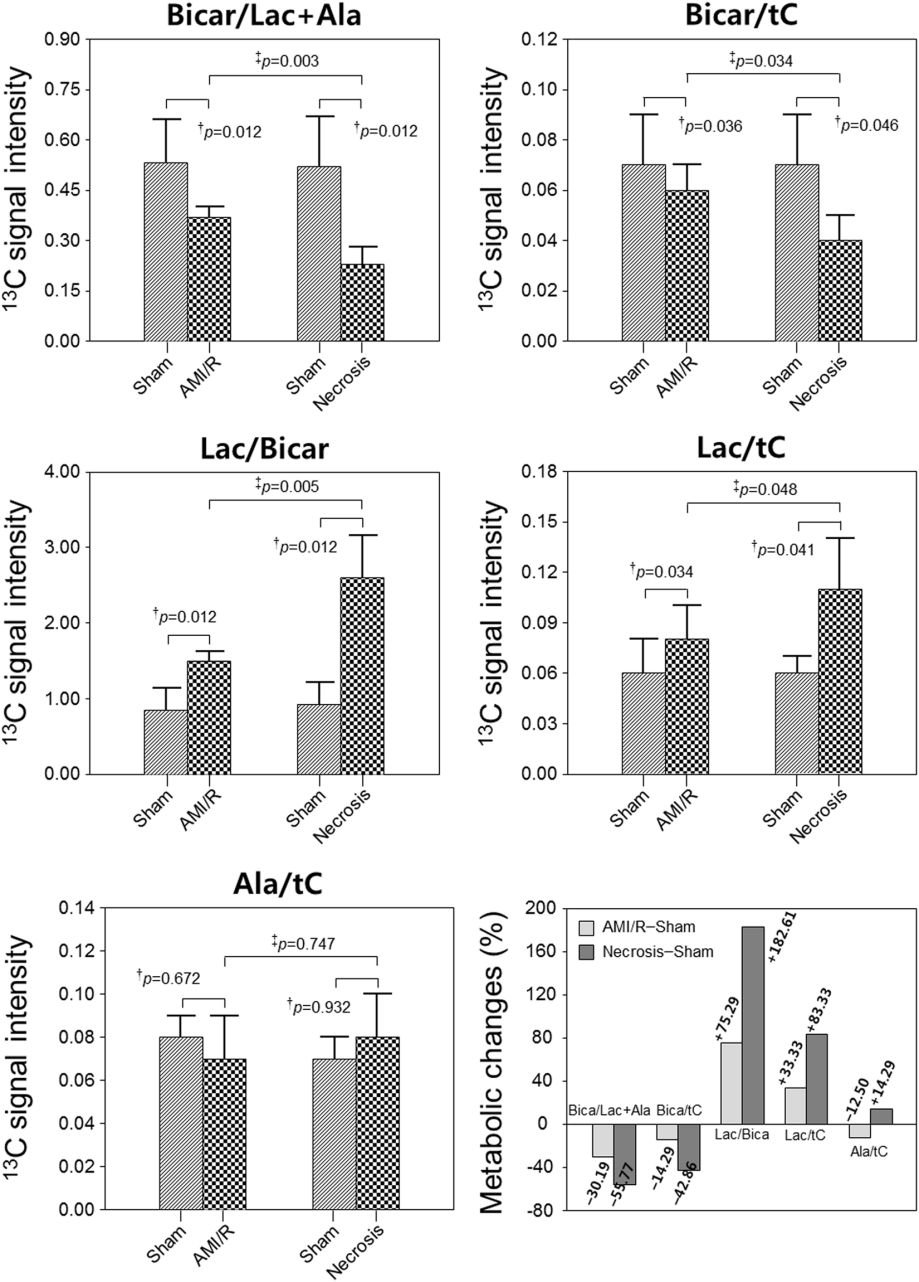
Comparison of the metabolite changes in sham-operated control, AMI/R, and necrosis groups. Changes (%) of metabolite level in AMI/R and necrosis groups relative to sham-operated control group. ^†^Significant difference (Wilcoxon signed-rank test; P < 0.05) between rats before and after AMI/R, and between rats before and after necrosis; ^‡^significant difference (Mann-Whitney U test; P < 0.05) between AMI/R and necrosis groups.

Figure 2c–e show the sum spectra reconstructed from the dynamic spectra covering short time periods from 16 to 60 s. Stacked spectra (Fig. 2f–h) and the corresponding dynamic intensity curves (Fig. 2i–k) depict the time-course of [1-^13^C] Lac, [1-^13^C] Pyr-hydrate, [1-^13^C] Ala, [1-^13^C] Pyr, and ^13^C Bicar after the administration of hyperpolarized [1-^13^C] Pyr. AMI/R and necrosis groups showed significantly higher ratios of [1-^13^C] Lac/Bicar and [1-^13^C] Lac/tC, and lower ratios of ^13^C Bicar/Lac + Ala and ^13^C Bicar/tC than those of the sham-operated control group (Fig. 3). Moreover, necrosis group showed significantly higher ratios of [1-^13^C] Lac/Bicar and [1-^13^C] Lac/tC, and lower ratios of ^13^C Bicar/Lac + Ala and ^13^C Bicar/tC than those of the AMI/R group (Fig. 3). However, there was no significant difference in the ratio of [1-^13^C] Ala/tC among the three groups.

**Figure 4:**
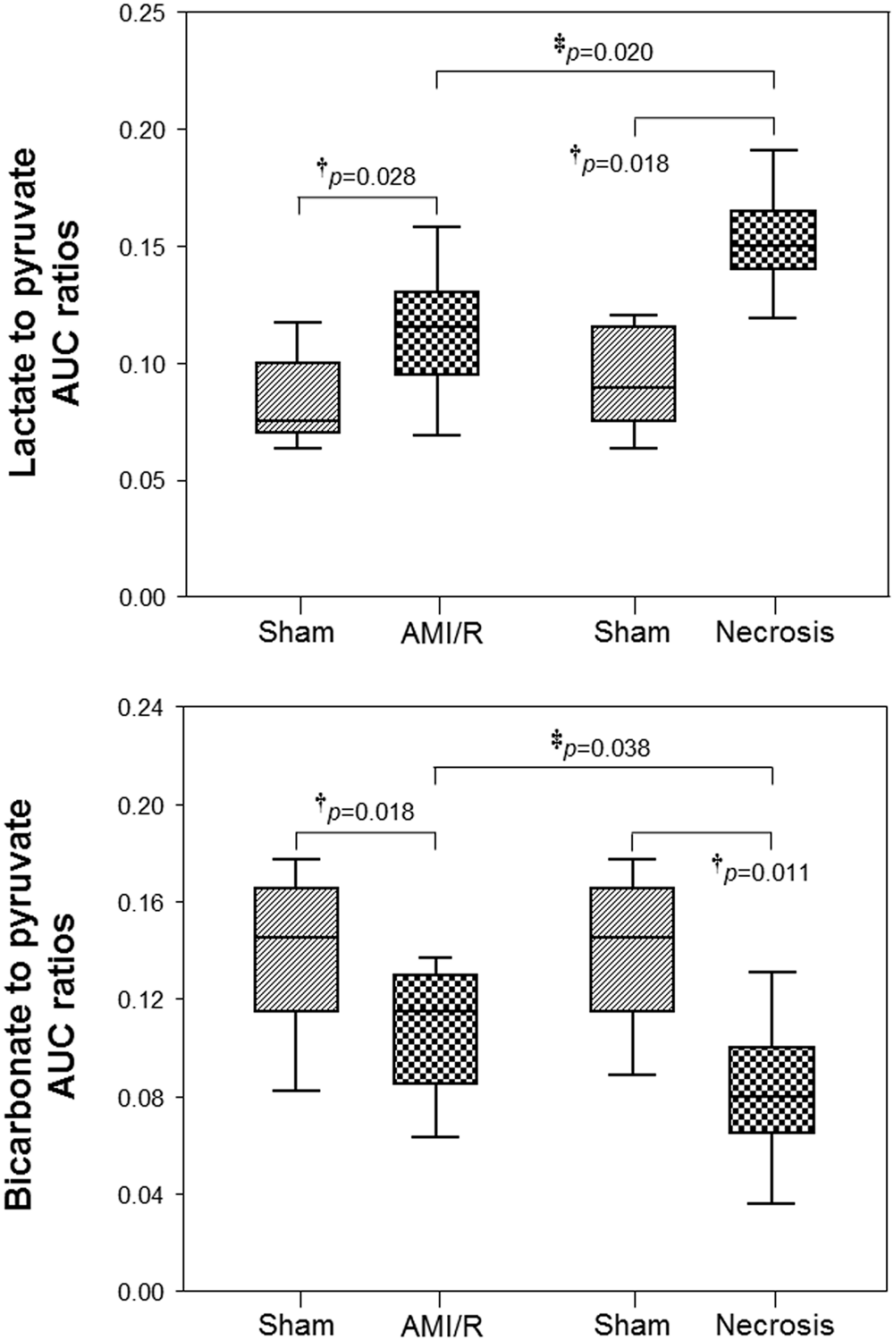
The AUC ratios of lactate/pyruvate and bicarbonate/pyruvate in sham-operated control, AMI/R, and necrosis groups. ^†^Significant difference (Wilcoxon signed-rank test; P < 0.05) between rats before and after AMI/R, and between rats before and after necrosis; ^‡^significant difference (Mann-Whitney U test; P < 0.05) between AMI/R and necrosis groups.

In addition, while the AUC ratio of Lac/Pyr was significantly higher in both AMI/R and AMI-induced necrosis groups than in the sham-operated control group (P < 0.05), the AUC ratio of Bicar/Pyr was significantly lower in both the AMI/R and AMI-induced necrosis groups (P < 0.05) (Fig. 4).

### *In vivo* measurement of pH and correlation with ^13^C-Lac and ^13^C-Bicar

**Figure 5:**
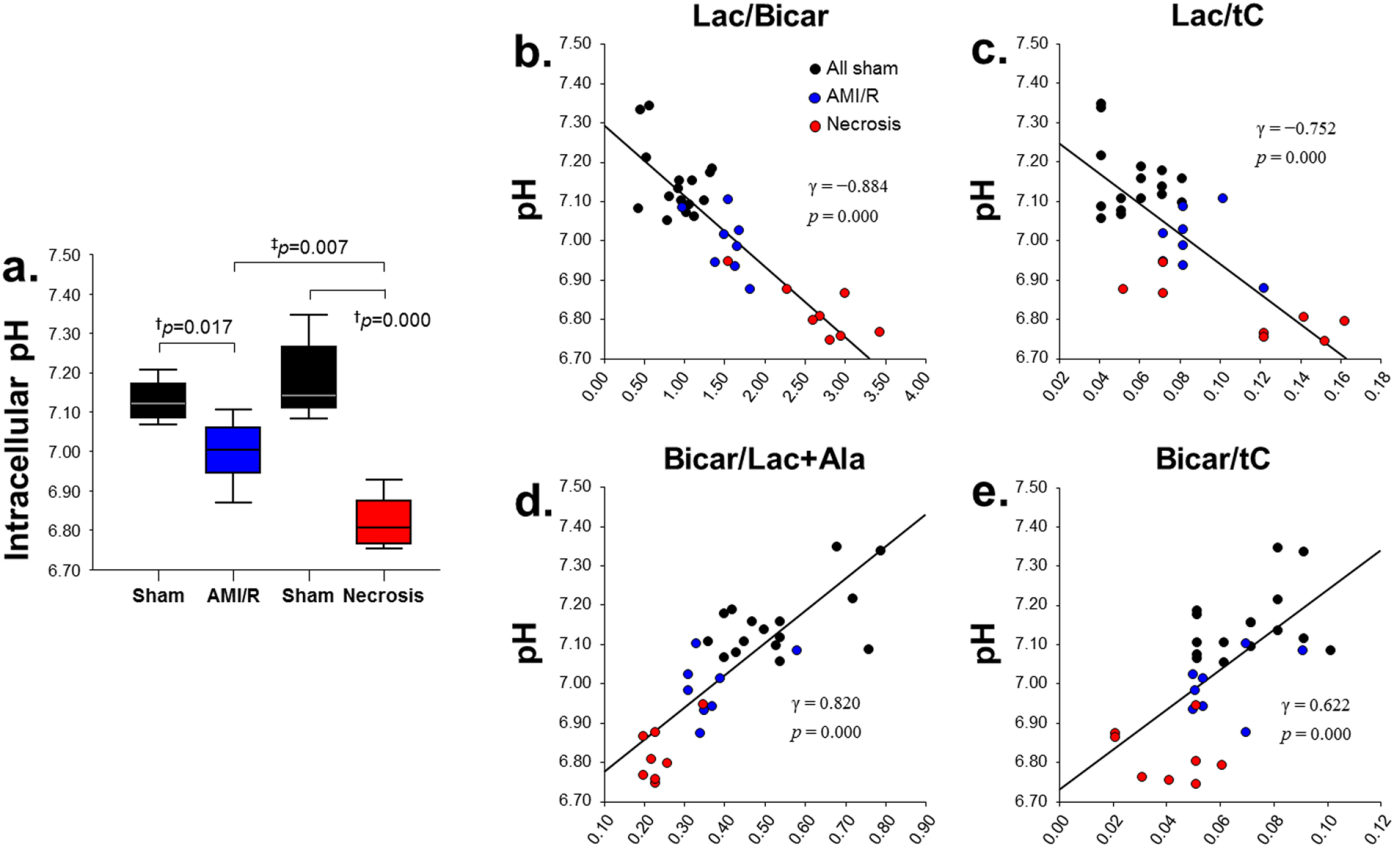
*In vivo* measurement of intracellular pH levels in sham-operated control, AMI/R, and necrosis groups (**a**) and the correlations between pH levels and the specific ^13^C-metabolic levels: [1-^13^C] lactate/bicarbonate (**b**), [1-^13^C] lactate/total carbon (**c**), ^13^C bicarbonate/lactate + alanine (**d**), and ^13^C bicarbonate/total carbon. (**e**) ^†^Significant difference (Wilcoxon signed-rank test; P < 0.05) between rats before and after AMI/R, and between rats before and after necrosis; ^‡^significant difference (Mann-Whitney U test; P < 0.05) between AMI/R and necrosis groups.

The intracellular pH level was decreased in the region of reperfused myocardium after AMI/R (Fig. 5a). Whereas levels of [1-^13^C] Lac/Bicar and [1-^13^C] Lac/tC were negatively correlated with pH levels (Fig. 5b,c), levels of ^13^C Bicar/Lac + Ala and ^13^C Bicar/tC were positively correlated with pH levels (Fig. 5d,e).

### ROC analysis for qualitative prediction of AMI/R and necrosis

**Figure 6:**
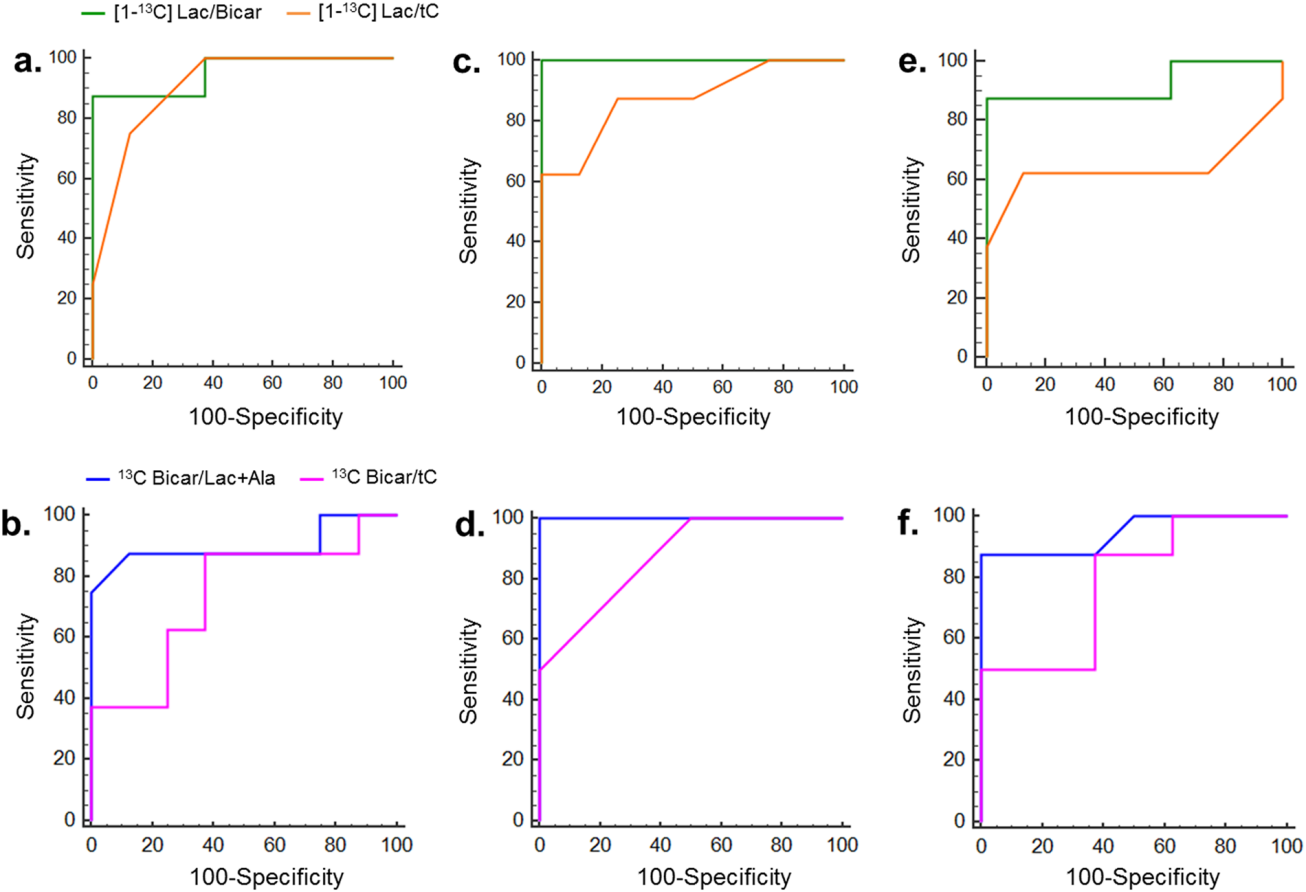
The ROC curves of [1-^13^C] lactate/bicarbonate (green), [1-^13^C] lactate/total carbon (orange), ^13^C bicarbonate/lactate + alanine (blue), and ^13^C bicarbonate/total carbon (pink) metabolite concentration ratios for the differentiation between sham-operated control and AMI/R groups (**a,b**), between sham-operated control and necrosis groups (**c,d**), and between AMI/R and necrosis groups (**e,f**).

Table 1 summarizes the results of the ROC analysis including the AUC, Youden index, sensitivity, and specificity values in sham-operated control, AMI/R, and necrosis groups. Also, Fig. 6 shows the ROC curves of Lac/Bicar (green), Lac/tC (orange), Bicar/Lac + Ala (blue), and Bicar/tC (pink) metabolite concentration ratios for the differentiation between three groups. Curves are compared between the AMI/R and sham-operated control goups (Fig. 6a,b), the necrosis and sham-operated control groups (Fig. 6c,d), and the AMI/R and necrosis groups (Fig. 6e,f).

**Table 1:** ROC curve analysis of qualitative prediction of AMI/R and necrosis.

Metabolites	AUC (95% CI)	Youden index	Sensitivity	Specificity
AMI/R – Sham				
Lac/Bicar	0.95 (0.77–0.99)	0.88	87.50	100.00
Lac/tC	0.91 (0.73–0.98)	0.69	100.00	68.75
Bicar/Lac + Ala	0.88 (0.68–0.98)	0.69	75.00	93.75
Bicar/tC	0.71 (0.51–0.88)	0.44	87.50	56.25
Necrosis – Sham				
Lac/Bicar	1.00 (0.86–1.00)	1.00	100.00	100.00
Lac/tC	0.87 (0.67–0.97)	0.63	62.50	100.00
Bicar/Lac + Ala	1.00 (0.86–1.00)	1.00	100.00	100.00
Bicar/tC	0.90 (0.70–0.98)	0.56	87.50	68.75
Necrosis – AMI/R				
Lac/Bicar	0.92 (0.68–0.99)	0.88	87.50	100.00
Lac/tC	0.64 (0.37–0.86)	0.50	62.50	87.50
Bicar/Lac + Ala	0.95 (0.71–0.99)	0.88	87.50	100.00
Bicar/tC	0.78 (0.51–0.95)	0.50	51.00	100.00

## Discussion

Acute changes in the cardiac metabolism of Pyr in association with intracellular pH levels were evaluated *in vivo* in a rat model of AMI/R using hyperpolarized ^13^C dynamic MRS. After occlusion of the LAD artery for 20 or 90 min, we injected hyperpolarized Pyr early during reperfusion of the artery at 15 min. In the rat model with AMI/R injury, the ratios of [1-^13^C] Lac/Bicar (+75.29%) and [1-^13^C] Lac/tC (+33.33%) were significantly higher than those in sham-operated control rats during early myocardial reperfusion, whereas [1-^13^C] Bicar/Lac + Ala (−30.19%) and ^13^C Bicar/tC (−14.29%) were significantly reduced (Fig. 3). Also, in the rat model with AMI-induced necrosis, the ratios of [1-^13^C] Lac/Bicar (+182.61%) and [1-^13^C] Lac/tC (+83.33%) were significantly higher than those in sham-operated control rats, whereas [1-^13^C] Bicar/Lac + Ala (−55.77%) and ^13^C Bicar/tC (−42.86%) were significantly reduced (Fig. 3).

Under anaerobic conditions, Pyr is metabolized to Lac by LDH. This generates a small amount of ATP and nicotinamide adenine dinucleotide, which is required for glycolysis^7^. The increased level of [1-^13^C] Lac with increased enzymatic acitivities of LDH and CK may be associated with cardiac damage. During reperfusion, the Lac-to-Bicar signal ratio was also significantly increased in the area at risk (AAR), indicating a reduction in oxidative metabolism with continuing anaerobic glycolysis^7^. In addition, the elevated Lac we observed is consistent with a previous finding from a study that investigated myocardial ischemia in the perfused heart^12^. Similar to an *ex vivo* study^25^, the Lac-to-Bicar ratio was increased in the AAR following a period of reperfusion. A study^26^ of the metabolites of I/R injury suggested that hypoxic and ischemic states could induce high levels of Lac when cells depend on anaerobic glycolysis. Under anaerobic conditions, the reaction catalyzed by LDH is as follows: Pyr + NADH + H^+^ ⇆ Lac + NAD^+^. In other words, as a robust indicator of the free cytosolic [NAD^+^]/[NADH] ratio, [1-^13^C] Lac/Pyr ratio in pathological states reflects altered metabolism^27^. Moreover, ischemic myocardium promotes an increase of glycolysis of stored glycogen in response to NADH that accumulates under hypoxic conditions^28^. Then, the opposing effects of increased NADH levels in ischemic tissue on oxidation lead to either an increase in [1-^13^C] Lac production or a decrease in ^13^C Bicar or both^17^. Myocardial ischemia reduces blood flow to provide adequate oxygen and can lead to hypoxia in various tissues. More severely, necrosis occurs within the hypoxic core of the ischemic area^29^. Although Lac produced by perinecrotic and hypoxic cells is cleared through the microvasculature, Lac can accumulate in the necrotic cavity due to the lack of a drainage route^30^. In the case of diffuse necrosis, a hyperpolarized ^13^C diffusion MRS study suggested that the ADC_Lac_/ADC_Pyr_ ratio might indicate the amount of cell death present^31^. Therefore, the significant change in hyperpolarized [1-^13^C] Lac signal detected soon after ischemia-reperfusion is presumably an indication of the AAR, reflecting reduced perfusion and nonviable myocardium.

On the other hand, the ^13^C Bicar signal is due to oxidative decarboxylation of [1-^13^C] Pyr and the pH-dependent equilibration of the resulting ^13^CO_2_ by carbonic anhydrase^16^. The Bicar-to-Lac ratio can be used in the rat model of ischemia-reperfusion to quantify the metabolic changes occurring immediately upon reperfusion^17^. In our study, the ^13^C Bicar signal was also measured within 15 min after 20 and 90 minutes of coronary artery occlusion, respectively, which might be an important factor in the sensitivity to differentiate metabolic changes in AMI/R injury and necrosis from normal myocardium. With an adequate oxygen supply, Pyr is metabolized in the mitochondria to CO_2_ and acetyl-CoA. CO_2_ is converted to HCO_3_^−^ to allow for CO_2_ transport and to maintain cellular pH^7^. Thus, the presence of HCO_3_^−^ reflects the formation of acetyl-CoA, which then enters the Krebs cycle. A decreased Bicar signal may be due to reduced flux through the Krebs cycle, and/or a switch to oxidation of fats or ketones^7^. More importantly, a positive correlation was revealed between the hyperpolarized ^13^C Bicar signal and pH level in both groups, whereas a negative correlation was found between the [1-^13^C] Lac signal and pH level (Fig. 5). From these findings, it is assumed that the intracellular pH level for acute metabolic acidosis induced by myocardial ischemia-reperfusion is associated with a reduction of ^13^C Bicar signal and an increment of [1-^13^C] Lac, which is considered a potential metabolic marker. It should be noted the hyperpolarized label could not be directly distinguished between metabolites located in intra- and extracellular spaces. Although trace amounts of ^13^CO_2_ might have diffused out of the myocardium and were subsequently hydrated to form HCO_3_^−^ by regulation of extracellular carbonic anhydrase, cardiac ^13^C spectra have indicated that HCO_3_^−^ is confined to the region of myocardium dominated by the intracellular space^11^.

Although animal numbers were low, we found a reasonably high overall accuracy of hyperpolarized ^13^C MRS to differentiate AMI/R and necrosis from sham-operated control, and necrosis from AMI/R at Bicar/Lac + Ala cutoff values and Lac/Bicar cutoff values indicated by the Youden index, whereas Bicar/tC and Lac/tC cutoff values had a low degree of accuracy. Consequently, the groups were discriminated relatively well from each other by use of the cutoff values.

Other metabolite levels, such as [1-^13^C] Ala, during early reperfusion remain normal immediately after injection of Pyr, and were not significantly different between two groups. Increased^32^ or decreased^11^ levels of [1-^13^C] Ala have previously been proposed as an additional indicator of myocardial viability following ischemia. A recent study^33^ using liquid chromatography-mass spectrometry metabolomics analysis also reported small changes in Ala levels in the heart during ischemia. However, no increase in Ala was observed in localized myocardial ischemia induced by LAD occlusion in intact mice. From these findings, it may be inferred that assessment methods for cardiac metabolism *ex vivo* and also in *in vivo* animal models is important in terms of reliability and reproducibility. A future study needs to modify the frequency response of the excitation pulse in order to allow detection of either preservation or reduction of Ala following ischemia, potentially indicating viability of the infarcted tissue.

Our study revealed that time-resolved hyperpolarized ^13^C Pyr cardiac metabolism is feasible, in which changes in the [1-^13^C] Lac and ^13^C Bicar signals reflect the metabolic acidosis at an earlier stage of reperfusion after acute myocardial ischemia and necrosis. However, the current study had some limitations. Despite the significant signal gains due to hyperpolarized MRS, the limited SNR of the detected metabolic products led to limited spatial resolution. Second, a follow-up study is needed to assess the long‐term effects of the use of medications for AMI/R injury on cellular metabolic alterations with perfusion abnormalities. Third, although we clarified the myocardial status to differentiate the groups accompanied by an alteration in pH, the ^13^CO_2_ signal is generally an order of magnitude lower *in vivo* compared with other metabolite ^13^C signals. Therefore, more research is needed to understand this important issue, based on a study^34^ which suggested that ^13^CO_2_ must be excited with larger flip angles to overcome SNR issues. Together with improved sensitivity of the ^13^CO_2_ signal, further experimental studies are needed to verify the pH values by other techniques, such as ^31^P MRS. Fourth, although liver metabolism could lead to statistically significant overestimation of cardiac lactate production^35^, liver lactate presaturation was not considered. Fifth, we did not measure the cardiac function of rats during the ischemic period. Also, the total Lac and Pyr pool sizes or cellular uptake of Pyr might differ among the groups. Instead, for quantification of the exchange rates, the AUC ratios of hyperpolarized [1-^13^C] Lac/Pyr and [1-^13^C] Bicar/Pyr were calculated and compared in the three groups.

## Conclusions

The current study demonstrated metabolite changes in rats with AMI/R and myocardial necrosis in connection with intracellular pH levels using *in vivo* hyperpolarized ¹³C MRS. Levels of [1-^13^C] Lac and ^13^C Bicar will be useful for real-time, non-invasive evaluation of the early stage of AMI/R and necrosis with reperfusion injury of the heart. These findings have potential application to real-time evaluation of cardiac malfunction accompanied by alterations of pH level and enzymatic activity.

## Data Availability

The data that support the findings of this study are available from the corresponding authors upon reasonable request.
